# Emotional “Contagion” in Piglets after Sensory Avoidance of Rewarding and Punishing Treatment

**DOI:** 10.3390/ani14071110

**Published:** 2024-04-04

**Authors:** Ye Zhang, Xuesong Yang, Fang Sun, Yaqian Zhang, Yuhan Yao, Ziyu Bai, Jiaqi Yu, Xiangyu Liu, Qian Zhao, Xiang Li, Jun Bao

**Affiliations:** 1 College of Animal Science and Technology, Northeast Agricultural University, Changjiang Road No. 600, Harbin 150030, China; zhangyeneau@163.com (Y.Z.); yangxuesong1204@163.com (X.Y.); sunfang276@163.com (F.S.); yinyuxingzhou@163.com (Y.Z.); yyh021023@126.com (Y.Y.); bzy050916@163.com (Z.B.); 18845721609@163.com (J.Y.); zhaoqian@neau.edu.cn (Q.Z.); 2 College of Life Science, Northeast Agricultural University, Changjiang Road No. 600, Harbin 150030, China; lily_liu1979@126.com

**Keywords:** pig, emotion, heart rate, animal welfare

## Abstract

**Simple Summary:**

In current pig farming, pigs are susceptible to stress-induced adverse emotions. Emotional contagion may propagate these emotions within a herd, potentially affecting overall welfare. Typically, treatments of individual pigs should be conducted separately from the group. Thus, after separation from treatment on an individual pig, the behavioral responses and heart rate changes of the companion pig were recorded to determine if the sensory avoidance was effective. It was found that peers were still affected by the treated pigs after sensory avoidance. Separation from treatment does not eliminate the effects of the treated pig on companion pigs, which can still be affected in post-treatment contact.

**Abstract:**

In the pig farming industry, it is recommended to avoid groups when treating individuals to reduce adverse reactions in the group. However, can this eliminate the adverse effects effectively? Piglets were assigned to the Rewarding Group (RG), the Punishing Group (PG), and the Paired Control Group (PCG). There were six replicates in each group, with two paired piglets per replicate. One piglet of the RG and PG was randomly selected as the Treated pig (TP), treated with food rewards or electric shock, and the other as the Naive pig (NP). The NPs in the RG and PG were unaware of the treatment process, and piglets in the PCG were not treated. The behavior and heart rate changes of all piglets were recorded. Compared to the RG, the NPs in the PG showed longer proximity but less contact behavior, and the TPs in the PG showed more freezing behavior. The percentage change in heart rate of the NPs was synchronized with the TPs. This shows that after sensory avoidance, the untreated pigs could also feel the emotions of their peers and their emotional state was affected by their peers, and the negative emotions in the pigs lasted longer than the positive emotions. The avoidance process does not prevent the transfer of negative emotions to peers via emotional contagion from the stimulated pig.

## 1. Introduction

The emotional state of animals is a crucial indicator for evaluating their well-being. High stocking densities in commercial farms may induce distress and fear, posing a significant challenge to animal welfare [1,2,3]. Animal welfare assessments should not only include indicators of negative emotions but also indicators of positive emotions to ensure that animals are in a positive state of welfare [4]. Behaviors such as playing and exploring are increasingly recognized as positive indicators and favorable predictors of animal welfare [5,6,7]. Food rewards such as popcorn [8], maple syrup [9], raisins, and apples [10,11] have been demonstrated to evoke positive emotions and behaviors in piglets. Earlier studies indicate that pigs can perceive information about the emotions of their peers, whether negative or positive [12,13]. Consequently, the arousal of either positive or negative emotions could influence the emotions of other pigs via emotional contagion. These effects might persist even after the stimulus ceases. It is necessary to comprehend this phenomenon for further investigation.

Emotional contagion is broadly defined as the emotional state-matching of a subject with another [14]. Behavioral adaptations in response to peers’ pain or distress are evident across various animal species such as mice [15,16], fowl [17,18], goats [5], and dairy cows [19]. Social animals can sense the emotional states of other individuals [20]. This raises the possibility that herd animals can be affected not only by direct stimuli but also by the emotional responses of their companions. Studies have demonstrated that pigs are sensitive to the emotional states of group members, and pigs not directly stimulated can experience emotional states induced by exposure to the emotional responses of their peers which have undergone painful experiences [21]. Following exposure to negative stimuli and restraint treatments, both of the treated pigs and their untreated companions show negative emotional states, despite the companions not experiencing the treatment directly [21,22,23]. Emotional contagion among domestic animals could significantly affect herds’ welfare. Conversely, harnessing this contagion to enhance positive group emotions, such as play behavior, can potentially elevate the overall emotional state and welfare of the animals.

Rewards and punishments are central to the elicitation of all emotional states; they determine the valence of emotions [24]. Both food rewards (such as syrup, apples, raisins, and chocolate) and punishment stimuli (including restraints, sudden noises, and thermal stimuli) are effective in inducing emotional states that persist after the stimulus [9,22]. Evidence of emotional contagion needs observers and demonstrators to have the same emotional valence, which provides ideas for this experimental design. Goumon and Špinka observed that untreated pigs exhibited fearful responses when a companion was restrained, demonstrating behaviors like freezing and reduced activity. They spent more time with their heads turned toward the restrained pigs, and approached and sniffed them frequently [21]. In Camerlink et al.’s study, demonstrators were treated positively or negatively, and observers showed more nose–nose contact behavior with pigs that received positive treatments and very little of that behavior with demonstrators that received negative stimuli [25]. Studies by Reimert et al. [10,11,26] also found that while companions showed different behavioral responses when their individuals were subjected to positive and negative treatments, the companion and the demonstrator pig were only visually compartmentalized, which did not eliminate the transmission of sound and odor information. In previous studies, observers directly witnessed or indirectly received information about the demonstrator’s processing, and it is impossible to exclude its influence on the emotion of the observing pig. Thus, it remains to be explored whether similar emotional responses occur when animals are unable to see and hear the processing of other individuals.

With standard management procedures such as tail docking [27] and castration [28] in the current swine industry, pigs are prone to negative emotions due to pain. Negative emotions affect welfare when emotional contagion occurs [29]. However, when avoiding the treatment process for the treated pigs, is it possible to reduce the negative effects on the peers? Based on previous research, the purpose of this trial was to explore comparisons of different pig roles under punishing or rewarding treatment. The behavior performance and the percentage change in heart rate were detected in this study to investigate whether the separation treatment could block the emotional spread of the differently treated pigs with their peers who were unaware of the treatment process.

## 2. Materials and Methods

### 2.1. Animals and Management

This study was conducted at the Shuangcheng Animal Farm, affiliated with Northeast Agricultural University, from April to June 2022. It involved 144 Yorkshire piglets, each four weeks old and of similar weight (9.27 ± 0.23 kg), distributed across twelve pens. Twelve pigs in each pen, weaned at 28 days of age, were housed in mixed-sex pens of 9.00 m^2^ (3.60 m × 2.50 m × 1.20 m, with 0.75 m^2^ per pig). The pens’ floors were lined with a 0.50 m layer of rice hulls for bedding, remaining unchanged throughout the study [30]. Water and standard pelleted feed were ad libitum available from a feeder and drinker. The feed composition included 3.30 MJ/kg of energy, 17.00% crude protein, 4.00% crude fiber, and 1.30% lysine. The pens benefited from natural ventilation and lighting, available from 10:00 to 14:00 daily, under conditions of 22–25 °C temperature and 67–73% humidity. A single individual managed the pig care and adherence to routine immunization protocols. The animal management practices complied with the Measures for Ethical Review of Science and Technology available in [31]. The entire experimental procedure was overseen by the Laboratory Animal Ethics Committee of Northeast Agricultural University (Approval number: NEAUEC2021 02 14).

### 2.2. Experimental Animal Grouping

The experiment was conducted at 9 weeks of age. The animals were tested in pairs, which consisted of same-sex pigs randomly selected from the same litter. A total of 72 pigs were selected from 12 pens (average weight of 25.07 ± 0.17 kg). Every six piglets were randomly selected from each pen and assigned to the three groups: a paired test unit with two piglets to the Rewarding Group (RG), the Punishing Group (PG), and the Paired Control Group (PCG), separately. One piglet in the RG and PG was randomly designated as the Treated Pig (TP) to receive reward or punishment treatment; the other one served as the Naive Pig (NP). All the piglets in the PCG were untreated. Each group was set up with 12 replicates: 6 for behavioral observation, 3 for heart rate testing, and 3 as the reserve pigs.

### 2.3. Treatments

**Figure 1:**
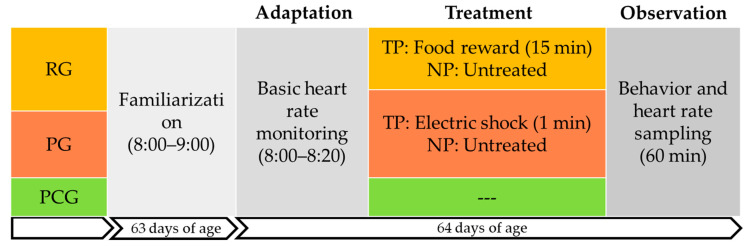
The flow of the experiment. The Rewarding Group (RG); the Punishing Group (PG); the Paired Control Group (PCG). Treated Pig (TP); Naive Pig (NP).

**Figure 2:**
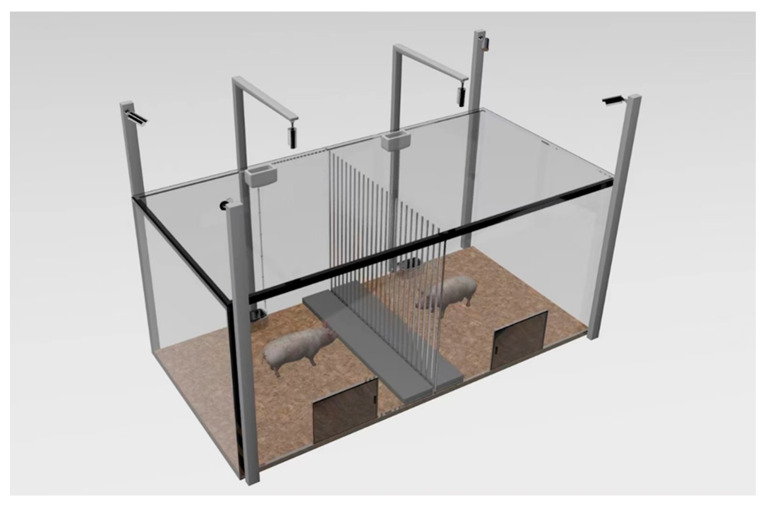
Three-dimensional structure of the test box. The test box is a rectangular test area (300 cm × 150 cm × 130 cm) surrounded by acrylic panels, which is separated into two identical square test areas with side lengths of 150 cm by a metal partition fence (150 cm × 130 cm, barrier diameter 0.5 cm, barrier spacing 5 cm) in the center. In addition, removable acoustic material-covered acrylic panels (the same size as the partitions) were placed at the center partition to fit the partitions, and laminated acoustic glass covered the top of each square test area to ensure that the other piglet was completely shielded from vision and hearing during the piglet treatment process. A platform with a height of 5 cm (150 cm × 25 cm × 5 cm) was set up in an area of 25 cm on each side of the partitions, and the other areas of the test box were paved with rice hulls with a thickness of 5 cm. In each square test area, a trough (20 cm × 17 cm × 10 cm) and a door (40 cm × 50 cm, made of the same material as the test box) were set opposite to each other, placed on both sides of the partition fence, 75 cm away from the partitions, and a water nipple was set up 10 cm near the side of the partitions next to the feed trough. Behavior-recording cameras were set up at 200 cm above each square test area and the corners of the test box.

At 63 days of age from 8:00–9:00 a.m., the piglets left their pens for 60 min to become familiar with the testing box. Every two piglets in the paired test unit were placed on opposite sides of the test box (as shown in Figure 1 and Figure 2). The acoustic panels were fitted to the partition and above the box to ensure that the NPs were not exposed to the visual and auditory information of the treatment process. The piglets were given ad libitum water during the test, and with no feed. After the observation, the piglets were returned to their home pens.

At 64 days of age, the test was conducted at 8:00 a.m. As shown in Figure 2, there were three periods in the PG and RG among the test: (1) the adaptation period was 20 min, which was used to record heart rate. (2) Subsequently, the TPs in the RG and PG were subjected to reward or punishment treatment during the treatment period, with food rewards (0.40 kg of feed, 0.10 kg of apples, and surplus food collected after 15 min) for fasting TPs in the RG, or with electroshock on the buttocks of the TPs in the PG (output voltage 8 V, consecutive stimulations three times, each interval 5 s, each duration 0.2 s). (3) Thereafter, the observation period came after the treatment period. The acoustic panel was removed when the treatment was completed to start the observation period; behavior and heart rate were recorded for one hour.

### 2.4. Behavioral Observation

Behavior was recorded with a surveillance system (Hangzhou Hikvision Digital Technology Co., Ltd., Hangzhou, China) and used focal sampling and continuous recording as the recording methods. Frequency (N) of nose–nose and nose–partition contact, escape attempts, and freezing, as well as duration (min) of proximity and exploring were recorded. Behavioral definitions are shown in Table 1.

### 2.5. Heart Rate Test

At the beginning of the adaptation period, the heart rate of each piglet was recorded using a heart rate monitor (LIFEDN ECG recorder, Shenzhen, China). It consisted of a flexible chest belt integrated with two electrodes and a radio transmitter for wireless data transmission, which can display the data at a mobile terminal. The position of the electrocardiographic patch was adjusted until stable heart rate data could be gathered, and the average heart rate of 11–20 min of the adaptation period was defined as the resting heart rate of the piglets in each group. During the observation period, the heart rate changes of the two pigs were continuously recorded, and the average heart rate was recorded every 5 min, totaling 12 average heart rates in one hour. The heart rate changes were described by a change in heart rate (%) [32], y indicates the average heart rate measured every 5 min. The formula is as follows:

$$Change\;in\;heart\;rate\;(\%)=\frac{y-y_{resting\;heart\;rate}}{y_{resting\;heart\;rate}}\times 100\%$$

### 2.6. Data Analysis

Data were statistically analyzed by SPSS 21 (SPSS Inc., Chicago, IL, USA). All data were examined for normal distribution using Kolmogorov–Smirnov test. The effects of different treatments or roles on pigs’ behavior were analyzed using two-way ANOVA. The statistical model of the two-way ANOVA was as follows: Y*_ij_* = *μ* + A*_i_* + B*_j_* + (AB)*_ij_* + *e*; Y*_ij_* is the target trait, *μ* is the overall mean, A*i* is the different treatment (rewarding or punishing, 2 levels), B*_j_* is the pig roles (TP or NP, 2 levels), (AB)*_ij_* is the interaction, and e is the random error. The behavioral data of pigs in the treatment groups and control group were analyzed using one-way ANOVA and Duncan’s multiple comparisons. The modeling was as follows: Y*_i_* = *µ* + A*_i_* + *e*; Y*_i_* is the target trait, *µ* is the ensemble average, A*_i_* is the different groups, and *e* is the random error. Curvilinear regression analyses were performed to determine the best-fit function for the heart rate of TPs or NPs and time, and the percentage change in heart rate of TPs and NPs, respectively. Results are presented as means ± SEM. A *p*-value of less than 0.05 was considered statistically significant.

## 3. Results

### 3.1. Behavioral Performance

**Figure 3:**
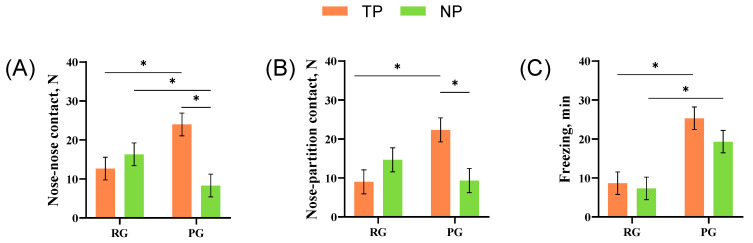
The effect of different treatments or roles on the behavior of pigs: (**A**) nose–nose contact; (**B**) nose–partition contact; (**C**) freezing. * Indicates significant differences between or within groups. The Rewarding Group (RG); the Punishing Group (PG).

As shown in Figure 3, in the PG, the TPs showed more frequent nose–nose and nose –partition contact than the NPs (*F*_1,20_ = 14.44, *p* < 0.01; *F*_1,20_ = 8.90, *p* < 0.01). In the TPs, there was significantly more nose–nose contact, nose–partition contact, and freezing (*F*_1,20_ = 7.56, *p* = 0.01; *F*_1,20_ = 9.36, *p* < 0.01; *F*_1,20_ = 16.61, *p* < 0.01) than in the RG. The NPs in the RG showed more nose–nose contact than in the PG (*F*_1,20_ = 10.58, *p* < 0.01). In contrast, the freezing was more frequent in the PG than in the RG (*F*_1,20_ = 14.44, *p* < 0.01). In addition, both the frequency of nose–nose contact (*F*_1,20_ = 5.25; *p* < 0.01) and nose–partition contact (*F*_1,20_ = 9.17; *p* < 0.01) were affected by the interaction effect of different stimuli (rewarding/punishing) and different roles (treated/naive).

**Figure 4:**
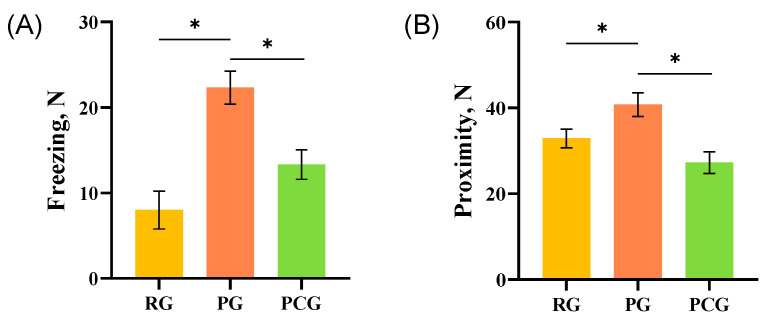
Behavior of pigs in the rewarding, punishing, and the paired control group. (**A**) freezing; (**B**) proximity; * Indicates significant differences between groups. The Rewarding Group (RG); the Punishing Group (PG); the Paired Control Group (PCG).

As shown in Figure 4, the pigs in the PG showed more frequent freezing and longer proximity than the RG and PCG (*F*_2,23_ = 7.38, *p* < 0.01; *F*_2,23_ = 13.62, *p* < 0.01). Furthermore, there were no differences in other behaviors of the pigs in the groups (all *p* > 0.05). 

### 3.2. Regression of Heart Rate

**Figure 5:**
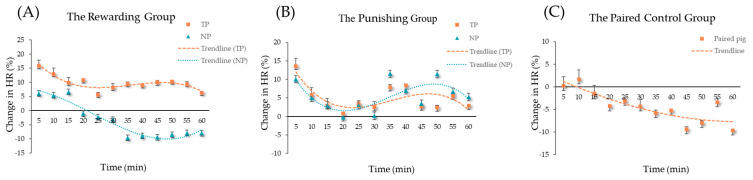
Regression of the heart rate of TPs and NPs with time. (**A**) The rewarding group; (**B**) The punishing group; (**C**) The paired control group.

The heart rate change (%) versus time of pigs in the RG and PG all conformed to a cubic regression curve, whereas it conformed to linear regression in the PCG (as shown in Table 2 and Figure 5). In the RG, the TPs had a higher percentage change in heart rate and the heart rate was always higher than the resting heart rate. The heart rate change (%) of NPs maintained a 5 min highest peak, then declined, as the heart rate was lower than the resting heart rate after 20 min (Figure 5A). The paired piglets in the PG showed a similar trend in the percentage change in heart rate and the heart rate was consistently higher than the resting heart rate (Figure 5B). As shown in Figure 5C, the heart rate change (%) of the piglets in the PCG decreased slowly and smoothly.

**Figure 6:**
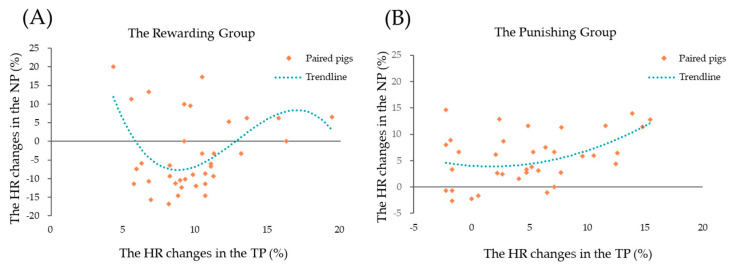
Regression of the heart rate of TPs and NPs. (**A**) The rewarding group; (**B**) the punishing group.

The results of the regression of heart rate change (%) curves of paired piglets in the RG and PG are shown in Table 3. In the RG, as the TPs’ heart rate increased, the NPs’ heart rate change (%) showed a negative–positive–negative correlation in that order (Figure 6A).

As shown in Figure 6B, the change (%) in the heart rate of paired piglets in the PG conforms to the quadratic regression curve. As the heart rate change (%) of the TPs increased, the heart rate change of the NPs showed a positive correlation.

## 4. Discussion

### 4.1. Reactions of Treated Pigs in Punishment and Reward Groups

The TPs in the punishing group (PG) showed an increased frequency of freezing. Freezing is a common response in the face of direct (perceived) threats in various species [33], suggesting fear, anxiety, alertness, and restlessness in piglets [34]. Reimert et al. [10,11] also found that pigs exhibited more freezing and escape attempts when treated negatively. Thus, we inferred that punishment triggered negative emotions according to the increase in freezing of TPs in PG. In addition, the heart rate findings for TPs in the PG corroborate the preceding discussion. The TPs showed the highest change in heart rate immediately after the punishment treatment and the heart rate was higher than the resting heart rate throughout. It seemed that punishment triggered emotional arousal in TPs and sensitivity to environmental changes. Previous studies have demonstrated an increase in heart rate among rats exposed to threatening environments [20]. Reimert [26] also observed an elevated heart rate in piglets during restraint treatment testing, compared to pre-test and post-test periods, suggesting an induced negative emotional state. However, a different point of view has been put forward: if an animal is less active due to negative emotions, and freezing for a long period to perceive its surroundings, a decrease in heart rate (called “fear bradycardia”) may also occur [35]. The phenomenon explains the reduced change in heart rate that occurs of the TPs in the PG, rather than the sustained maintenance of a higher heart rate. Furthermore, human studies have revealed that negative emotions, such as sadness, are associated with a decreased heart rate [19]. In summary, it is inferred that the electrical shock, as a form of negative treatment, elicited negative emotions in the TPs.

Studies have shown that providing pigs with popcorn [8], raisins, and apples [10,11] all caused a positive change in emotion. In this study, apples and feed were provided for fasting TPs as positive treatments for the rewarding group (RG). This study found reduced freezing of the TP in the RG, which might also be a sign of a positive emotional state. However, the difference between RG and the paired control group (PCG) was not significant, perhaps because the treatment did not supply a sufficient reward, or maybe the emotion was not strong enough and not long-lasting. Thus, the rewards reduced the occurrence of freezing in pigs, but the increase in exploring that characterized positive emotions remained insignificant. However, the heart rate of the TP in the RG was higher than the resting heart rate, implying emotional arousal. The heart rate illustrated a decreasing trend in the 20 min after the reward treatment, but the fluctuation of the change was not significant after 20 min. The results suggest that the positive emotions triggered by the reward treatment maintained a high arousal level only in the first 20 min, and after that, the arousal level of positive emotions may have become lower, and the heart rate was relatively stable, which may also be the reason for the insignificant increase in the positive exploring behaviors compared to that of the PCG. Research indicates that negative emotions may be easier to experimentally induce than positive emotions, and that they may be more salient in their expression than positive emotions [36]. In all, both the reduction in freezing and the change in heart rate indicate that rewards might awaken positive emotions in piglets, but that the extent of arousal may diminish over time.

### 4.2. Emotional Contagion and Social Interactions between Treated and Naive Pigs

In previous studies, observers could gather information about the demonstrator’s treatment process via visual, auditory, olfactory, and body contact [10,26], which affects the observer directly. In this experiment, the NPs in the PG showed more freezing after the treatment on the TPs although the NPs did not see or hear the treatment process when the TPs were treated. Animals showed frequent freezing as a sign of fear when companions were in distress [37,38]. This is consistent with Goumon’s findings, in which pigs engaged in more freezing behavior when confronted with stress-treated companions [21]. In addition, both in the rewarding and punishing groups, the heart rate of treated and naive pigs was synchronized over time. Furthermore, the heart rate of NPs in the PG was almost consistently higher than the resting heart rate during the observation period and showed significant fluctuations. Whereas, the heart rate of NPs in the RG was higher than the resting heart rate for the first 20 min, after which it gradually decreased and stabilized. Also, the NPs in the RG showed less freezing, which was similar to TPs and lower than PG. This shows that the NPs in the RG are in a relatively positive state compared to the NPs in the PG. In summary, emotional contagion occurs between treated and naive pigs, and the negative emotions of the naive pigs in the punishing group last longer than the positive emotions of the naive pigs in the rewarding group.

Proximity seeking can be conceived of as a social form of risk assessment that benefits the approaching observer: a propensity that leads to the collection of valuable information about potential dangers [36]. Moreover, animals are not only sensitive to pain and sometimes act to alleviate pain from themselves, but also emotionally support the animal in pain [39]. Goumon’s study found that pigs actively approached their companion when observing a negatively treated mate and spent more time looking at the pairmate. It may be that the observer provides social support to the demonstrator [21]. In the current study, the NPs in the PG approached the center partition more, possibly to gather information or to support the TPs. However, the NPs in the PG showed less nose–nose contact which might be threat-avoidance behavior in response to the TPs’ negative emotions. When mice witnessed other individuals suffer electric shocks, they hid in shelters when given the choice and eventually escaped from the shocked demonstrator [36]. Fear can be transmitted through social interaction, a mechanism that is an adaptive manifestation that promotes defensive, avoidance behaviors to adapt to potentially dangerous situations in the environment [40]. When animals are exposed to novel or unexpected stimuli, they show fear (avoiding potential threats) and curiosity (gathering information and reducing uncertainty) [41]. Also, approach and sniffing may play a role in cue acquisition [8]. In the RG, the NPs might gain information about reward treatment via social interaction; in addition, it has been investigated that the social interaction of pigs with each other in non-threatening situations may be social play [42]. Consistent with this experiment, Camerlink et al. found that by subjecting demonstration pigs to positive and negative stimuli, observers showed more nose–nose contact with pigs receiving positive treatments; in contrast, pigs receiving negative stimuli seldom performed the behavior. This suggests that pigs may recognize the emotional state of their peers and change their social behavior toward their peers [25]. Therefore, the frequent interaction of the NPs in the PG might be induced by TPs’ positive emotions. Or, it might be to gather information from their peers, which also indicated that the emotions of NPs were not negative at least. Pigs are sensitive to the emotions of their peers, and the NPs try to match the emotional state of the TPs regardless of any treatment the TPs undergo.

## 5. Conclusions

After the sensory avoidance of the negative treatment processes, naive piglets that were unaware of the treatment process showed more freezing and approach intention but less body contact with their peers. Whereas with the reward treatment, the naive piglets showed more nose–nose contact and fewer freezing behaviors with their peers. In pig farming, separation from treatment cannot eliminate the influence on peers who were not treated. Piglets could also perceive the emotional information from their peers, and their emotional state was affected by their groupmates.

## Figures and Tables

**Table 1:** Definitions of observed behaviors.

Behavior	Definition
Nose–nose contact (N)	Touching the nose of another pig with the rooting disc (initiated by different pig roles)
Nose–partition contact (N)	Touching the central partition with the rooting disc
Proximity (min)	Head within 25 cm of the center partition
Exploring (min)	Sniffing, nosing, or rooting the rice hull and the walls of the pen in the rice husk area
Escape attempts (N)	Pig jumps in the air or against the wall or door of a compartment
Freezing (N)	Standing motionless with whole body and head fixed

Note: Behavioral indicators refer to Reimert, and Goumon and Spinka [21,23].

**Table 2:** Regression analysis of heart rate change (%) versus time in different groups of piglets.

Equation	Role	R^2^	F	df_1_	df_2_	*p*-Value
The rewarding group						
cubic	TPs	0.59	15.31	3	32	<0.01
cubic	NPs	0.36	6.06	3	32	<0.01
The punishing group						
cubic	TPs	0.26	3.65	3	32	0.02
cubic	NPs	0.31	4.77	3	32	<0.01
The paired control group						
linear	paired pig	0.24	22.07	1	70	<0.01

**Table 3:** Curvilinear regression of heart rate change (%) for the TPs and NPs.

Equation	R^2^	F	df_1_	df_2_	*p*-Value
The rewarding group					
cubic	0.257	3.685	3	32	0.02
The punishing group					
quadratic	0.207	4.319	2	33	0.02

## Data Availability

The data presented in this study are available on request from the corresponding author.
